# Left ventricular remodeling following percutaneous edge-to-edge repair using Mitraclip

**DOI:** 10.1186/1532-429X-17-S1-P343

**Published:** 2015-02-03

**Authors:** Bernard P Paelinck, Tom Vandendriessche, Dina De Bock, Paul M Parizel, Inez E Rodrigus, Claeys J Marc

**Affiliations:** 1 Cardiac Surgery, Antwerp University Hospital, Edegem, Belgium; 2 Cardiology, Antwerp University Hospital, Edegem, Belgium; 3 Radiology, Antwerp university Hospital, Edegem, Belgium

## Background

Percutaneous edge-to-edge repair with MitraClip has been recommended as an alternative to conventional mitral valve repair in symptomatic severe mitral regurgitation at very high surgical risk. Data on the impact of MitraClip on left ventricular volumes are scarce. Therefore we aimed to assess left ventricular remodeling following MitraClip.

## Methods

Consecutive patients scheduled for MitraClip were prospectively studied. Patients with pacemaker or ICD were excluded. Transthoracic echocardiography and CMR at 1.5 Tesla were performed before and 6 months after MitraClip procedure. Left ventricular volumes were measured using transthoracic echocardiography (biplane Simpson's method) and short-axis SSFP CMR images covering the left ventricle. Mitral insufficiency was graded using color Doppler flow mapping (grade 1-4).

## Results

CMR was performed in 16 patients with symptomatic severe (grade 3 or 4) functional mitral regurgitation (median 73 years (range 45-87 years), M/F: 7/9, left ventricular ejection fraction (EF), 31% (20-73%), logistic EuroSCORE 23% (10-35%)). MitraClip reduced mitral regurgitation (residual grade 1 in 6 patients, grade 2 in 7 patients and grade 3 in 1 patient), but failed in 2 patients (residual grade 4). MitraClip caused intracavitary metallic artefacts at the base of the heart, but did not impede endocardial border definition. No change in left ventricular volumes was measured following successful MitraClip implantation (enddiastolic volume before MitraClip: 221 ml (102-334 ml) versus following MitraClip: 215 ml (79-284,5 ml) NS; endsystolic volume: 154 ml (31-248 ml) versus 145 ml (40-240 ml) NS and ejection fraction: 31% (20-73%) versus 32% (16-61%) NS). Negative remodeling occurred in both patients with MitraClip failure (enddiastolic volume before MitraClip:330 and 250 ml versus following MitraClip: 370 and 260 ml respectively, endsystolic volume:224 and 184 ml versus 258 and 213 ml respectively). There was a high correlation between biplane Simpson's method and CMR assessed left ventricular enddiastolic (r=0.93, P<0.001) and endsystolic (r=0.95, P<0.001) volumes.

## Conclusions

CMR allowed precise assessment of left ventricular volumes. Despite reduction of mitral regurgitation, no change of left ventricular volumes was measured at 6 months follow-up.

## Funding

N/A.

